# Flexible Patched Brain Transformer model for EEG decoding

**DOI:** 10.1038/s41598-025-86294-3

**Published:** 2025-03-29

**Authors:** Timon Klein, Piotr Minakowski, Sebastian Sager

**Affiliations:** 1 Department of Mathematics, Otto-von-Guericke University Magdeburg, 39106 Magdeburg, Germany; 2 Max Planck Institute for Dynamics of Complex Technical Systems, 39106 Magdeburg, Germany

**Keywords:** EEG decoding, Machine learning, Attention-based model, Motor imaginary dataset, Learning algorithms, Network models, Neural decoding

## Abstract

Decoding the human brain using non-invasive methods is a significant challenge. This study aims to enhance electroencephalography (EEG) decoding by developing of machine learning methods. Specifically, we propose the novel, attention-based *Patched Brain Transformer* model to achieve this goal. The model exhibits flexibility regarding the number of EEG channels and recording duration, enabling effective pre-training across diverse datasets. We investigate the effect of data augmentation methods and pre-training on the training process. To gain insights into the training behavior, we incorporate an inspection of the architecture. We compare our model with state-of-the-art models and demonstrate superior performance using only a fraction of the parameters. The results are achieved with supervised pre-training, coupled with time shifts as data augmentation for multi-participant classification on motor imagery datasets.

## Introduction

Decoding the human brain with machine learning methods has the potential to unlock a deeper understanding of neurological activities and diagnoses. A particularly suitable, non-invasive method to collect data is electroencephalography (EEG), which measures the brain’s electrical activity using electrodes placed on the scalp. Known for its high temporal resolution, EEG captures rapid changes in brain activities, which makes it valuable for both research and clinical examinations. This work focuses on decoding imagined movements (motor imagery) as a proxy for classifying brain states more generally with EEG signals apart. The proposed methods can furthermore be applied to related tasks such as emotion recognition^1^, classification of focus states^2^ and control for robotics or games^3,4^. EEG based diagnosis serves as the gold standard for detecting epilepsy^5^ and is also employed in recognizing various neurological disorders. Although there are many potential applications for decoding EEG signals, it remains a challenging task for both neuroscience and deep neural networks^6^. This is namely due to the variability between participants and even between different recording sessions from the same subject, for example, due to variations in baseline brain activities. Moreover, publicly available data is limited. Typical datasets consist of 10–100 subjects with approximately 200 labeled trials per subject^7,8^.

Previous research on decoding EEG signals mainly focuses on the individual subject-level classification with shallow convolutional neural networks (CNN)^9,10^. However, they are unable to improve performance as the amount of data scales^10,11^. Recent Transformer based approaches^12–14^ for EEG decoding follow the successful recipes for training foundation models in natural language processing (NLP)^15^ and computer vision^16^ by scaling the model size, the amount of training data in pre-training, and the compute time. Notably, only the size of the CNN tokenizer for these Transformers exceeds the size of previously used CNN-based models. To train large models effectively, a significant amount of diverse and high-quality data is required to capture patterns and generalize effectively. The scarcity of such data poses a bottleneck for large models in brain-computer interface (BCI) applications.

In this study, we propose the *Patched Brain Transformer* (PBT), an efficient adaptation of the Vision Transformer (ViT)^17^ designed for decoding EEG signals. Our model is designed to be adaptive regarding the number of EEG channels and the signal length over time, enabling it to process multiple datasets with varying EEG sensor arrangements. The raw EEG data are tokenized channel-wisely into fixed-size patches and then linearly embedded. Together with a classification token and added positional encoding, the sequence is fed to the Transformer. The final classification is performed by a linear layer on the specially designated $[c \,ls]$-token.

We demonstrate that a model with a carefully chosen size, combined with a well-engineered training process, achieves state-of-the-art results in EEG-based classification of imagined movement. Contrary to prior studies, our research demonstrates the feasibility of applying a pure Transformer to linearly embedded patch-based raw EEG signals. This reduced approach minimizes the number of required hyperparameters. In our experiments, we investigated the combination of data augmentation, regularization, and pre-training for training the PBT. Supervised pre-training is demonstrated to be more effective than its self-supervised counterpart. This underscores the significance of our proposed approach, setting the stage for the detailed exploration and analysis presented in the subsequent sections of this paper.

## Related work

The predominant architectures for EEG decoding are convolutional neural networks^9,10,18^, inspired by the field of image recognition^19^. CNN models deploy separate convolutions along the time dimension to learn representations in frequency space, followed by depth-wise convolution to extract spatial feature maps from the locations of electrodes on the scalp. EEGNet^11^ was designed to learn from a limited amount of data using a small number of trainable parameters and a few non-linear activation functions. This is particularly important given the limited training examples in currently available public EEG datasets. It is known that scaling the amount of training data leads to saturation with no increase in performance^10,11^. Additionally, CNNs are constrained by their fixed input structure.

An approach to overcoming the fixed input structure of CNNs is to employ Graph Neural Networks (GNNs). Most GNNs applied to EEG decoding are based on Graph Convolutional Neural Networks (GCNNs)^20,21^. By incorporating the relationships between individual sensors, modeled as a graph, these networks account for the complex structure of the human brain while retaining CNN layers as the backbone of the GCNN^22,23^. However, in GCNNs, the input dimensions are fixed. The graph connecting the sensors remains unchanged across all samples and even across different subjects.

In contrast to the shallow models commonly employed in EEG decoding, state-of-the-art models^24^ in Natural Language Processing (NLP) are notably larger, with a trend towards further scaling^15^. Pre-training is fundamental for the success of these massive NLP models, involving self-supervised learning on an extensive corpus of text. Common pre-training tasks include bidirectional token reconstruction, as proposed in the BERT paper^25^, and predicting the next word, as applied in the GPT line of models^26^. Inspired by the success of Transformers in NLP, researchers explored their adaptation to tasks beyond text processing. For instance, wav2vec 2.0^27^ is a model explicitly designed for speech recognition. It incorporates a CNN to generate latent representations from raw audio waves, serving as input tokens for the Transformer encoder.

BENDR^12^, the pioneering Transformer model designed for EEG decoding, introduced this combination of CNN and Transformer architectures for EEG decoding and has since been adopted elsewhere^13,14,28^. These models extend wav2vec 2.0^27^, initially designed for a single input sensor, to accommodate multiple input sensors. Acknowledging BERT’s approach in NLP, Kostas et al.^12^ and Chien et al.^29^ explored self-supervised pre-training for BENDR, where the convolutional stage generates a sequence of representations summarizing the input, and contiguous spans of this sequence are masked before being processed by the Transformer. The training optimizes a loss function that ensures the Transformer’s output for a masked position aligns closely with the unmasked input, using cosine similarity and a set of distractor negatives, while regularizing activations to prevent them from growing too large.

In contrast to BENDR, which applies convolutions in the spatial dimension, ATCNet^13^ combines temporal and spatial convolutions as a tokenizer and is particularly optimized for Motor Imagery (MI) classification. LaBraM^14^ achieved flexibility in the number of input channels by applying convolutions in the temporal dimension. Furthermore, LaBraM proceeds with vector-quantized neural spectrum prediction as self-supervised pre-training. Interestingly, in their study, Kostas et al.^12^ demonstrated that after pre-training on the combined tokenizer and Transformer, the entire Transformer can be substituted with a single linear layer to improve decoding results. Note that the resulting architecture consists only of the CNN tokenizer and the additional linear layer.

In computer vision, the use of combined CNN and Transformer architectures is limited^30^. However, the breakthrough for Transformers in computer vision was realized with the ViT^17^. This model uses the Transformer directly on image patches, resulting in a state-of-the-art approach that surpassed CNNs in image recognition. The success of this patch-based approach has been demonstrated in various computer vision tasks^31,32^.

Following this approach, Yang et al.^33^ proposed BIOT. The model transforms the raw EEG signals with the Fast Fourier Transform (FFT) into discrete frequency features as inputs to a modified ViT. This transformation to the frequency space sacrifices temporal resolution and increases the already high input dimension. To address the challenge of high input dimensionality, the training pipeline incorporates Low-Rank techniques^34–37^, along with supervised pre-training and self-supervised pre-training, achieved through the reconstruction of manipulated inputs using contrastive loss^38^.

Similar to BIOT, our model is inspired by ViT. However, we propose a linear embedding combined with a learned positional embedding. Our experiments show that these improvements accelerate training, thereby reducing the number of training epochs. More importantly, we demonstrate superior decoding results.

## Model

**Fig. 1:**
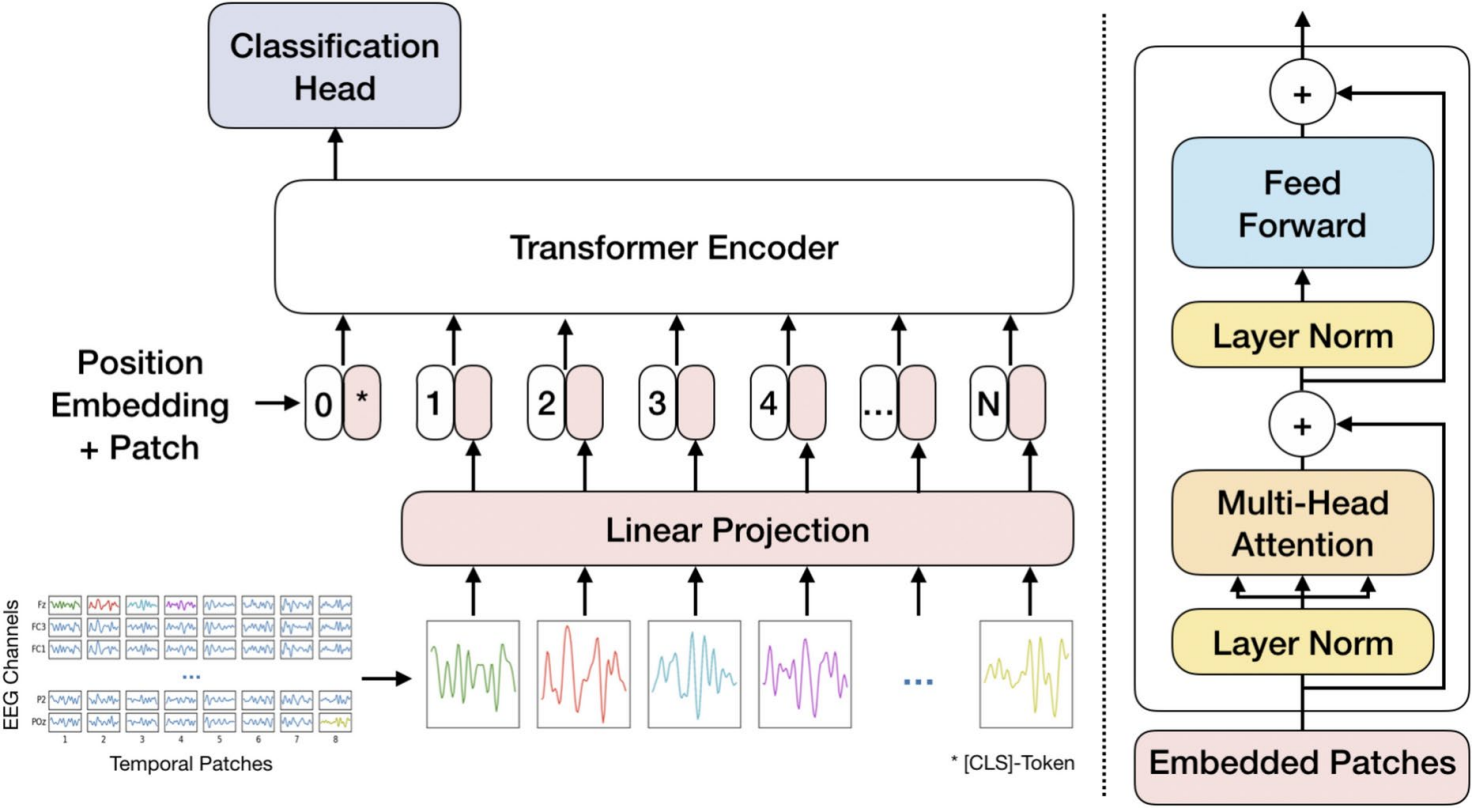
**Left:** Illustration of the Patched Brain Transformer model architecture. **Right:** Sketch of the Transformer Encoder Layer.

Our model is called Patched Brain Transformer (PBT), where the architecture is inspired by the Vision Transformer^17^, as illustrated in Fig. 1. EEG trials are partitioned into fixed-size patches along the channel dimensions, and patches are then linearly embedded. A classification token is inserted at the beginning of the sequence for further processing. To introduce positional dependence between patches, we add a trainable positional embedding to each of them. Subsequently, the token sequence is fed into the Transformer encoder. The final classification is executed by a linear classification head, exclusively on the classification token.

### Tokenizer

Pre-processed EEG signals are represented in a matrix, $X \in \mathbb {R}^{C \times T}$, where *C* denotes the number of EEG channels and *T* represents the time dimension. In this context, *T* corresponds to the product of the length of a trial in seconds and the frequency of the recording. The tokenizer transforms the matrix *X* into a sequence of patches denoted as $X_{\text {P}} \in \mathbb {R}^{N \times D}$, where *D* represents the patch dimension, a model hyperparameter. The number of patches is $N = C \lfloor \frac{T}{D} \rfloor$. It is important to note that when applying time shifts as part of data augmentation, the number of patches is further decreased, resulting in $N = C \lfloor \frac{T - T_{\text {aug}}}{D} \rfloor$, where $T_{\text {aug}}$ represents the time shift hyperparameter. Consequently, the number of training trials increases.

### Linear projection

The patches are projected into a latent space using a linear layer $X_{\text {E}} = X_{\text {P}} W_{\text {E}}^T,$ where ${W_{\text {E}} \in \mathbb {R}^{d_{\text {model}} \times D}}$. The model dimension is chosen as $d_{\text {model}}=2D$.

### Embedding and classification-token

A classification token, $[c \,ls] \in \mathbb {R}^{1 \times d_{\text {model}}}$, is preceded to the patch sequence, see BERT^25^. Furthermore, to address the positional invariance of the Transformer encoder, a learnable positional embedding, $W_{\text {pos}} \in \mathbb {R}^{(N+1) \times d_{\text {model}}}$, is added to each patch,

1 $$\begin{aligned} X_{\text {pos}} = \begin{bmatrix} c\,ls \\ X_{\text {E}} \end{bmatrix} + W_{\text {pos}}. \end{aligned}$$

### Transformer encoder

The Transformer encoder layers, built upon the original Transformer architecture^24^, consist of two main elements: the multi-head self-attention (MHSA) block and a feed-forward network (FFN). Each of these blocks has a parallel residual connection^39^. In contrast to the original Transformer, layer normalization (LN)^40^ is positioned within the blocks that are bypassed by the residual connections^41,42^,

2 $$\begin{aligned} X_{\text {MHSA}}&= {\text {MHSA}}({\text{LN}}(X_{\text {pos}})) + X_{\text {pos}}, \end{aligned}$$

3 $$\begin{aligned} X_{\text {FFN}}&= {\text {FFN}}({\text{LN}}(X_{\text {MHSA}})) + X_{\text {MHSA}}. \end{aligned}$$

### Classification head

The classification is performed on the processed classification token by a linear layer

4 $$\begin{aligned} y = {\text {softmax}} \left( [c\,ls] W^T_{\text {class}} + b_{\text {class}}\right) , \end{aligned}$$

where $W_{\text {class}} \in \mathbb {R}^{N_{\text {class}} \times d_{\text {model}}}$, $b_{\text {class}} \in \mathbb {R}^{1 \times N_{\text {class}}}$ and $N_{\text {class}}$ denotes the number of classes. In a supervised setting, all tokens are dropped, except for the classification token.

## Data description

In this study, we employ EEG recordings of human brains, separated into datasets for pre-training and fine-tuning. We choose the pre-training datasets with a recording procedure consistent with the actual target datasets. Consequently, both pre-training and fine-tuning trials consist of the presentation of a fixation cross accompanied by an acoustic warning tone. Following a brief interval, a cue appears on the screen instructing participants to imagine movement for a few seconds. Only the part of the EEG recordings in which the participants experienced the imaginary movement is included in our analysis.

### Datasets for pre-training

We aim to pre-train on recordings from a diverse group of subjects, including participants of different genders and ages. The recordings from 240 subjects are equally distributed between participants identified as male or female to ensure the learning of general representations.

The pre-training dataset consists of five datasets, totaling 42,256 trials. Detailed information about the included data sets is provided in Table 1. Datasets with a recording frequency different from 250 Hz are resampled to this frequency. In the case of supervised pre-training, the labels from the different datasets are merged, resulting in approximately equal distribution across the five classes.

**Table 1:** Summary of datasets.

	Dataset	Trials	Subjects	Channels	Classes
Pre-training	AlexMI^43^	480	8	16	Right hand, feet, rest
	BNCI2015004^8^	1420	9	30	Right hand, feet
	Cho2017^44^	9880	49	64	Left hand, right hand
	Lee219 MI^45^	10800	54	55	Left hand, right hand
	Physionet MI^46^	19676	109	64	Left hand, right hand, feet, rest, both hands
	$\Sigma$	42256	240		
Fine-tuning	BCI Competition IV 2a^47^	5184	9	22	Left hand, right hand, feet, tongue
	BCI Competition IV 2b^48,49^	6520	9	3	Left hand, right hand

### Data for fine-tuning

As the target classification dataset, we chose two datasets from BCI Competition IV^7^. The trials, each lasting four seconds, are captured at a sampling rate of 250 Hz, yielding 1000 data points per channel.

The BCI Competition IV 2a dataset^47^ features nine subjects, each contributing 288 trials per session. Trials are balanced across the four classes: left hand, right hand, foot, and tongue. The sensor arrangement includes 22 EEG channels. Participants undergo two separate recording sessions on different days: one designated for training and the other for evaluation.

The BCI Competition IV 2b dataset^48,49^ encompasses EEG data from nine subjects, with each subject accounting for 680 to 760 trials. These recordings consist of three EEG channels and pertain to two classes: left hand and right hand. Both classes have the same number of trials. The set of recordings consists of five sessions per subject, such that sessions zero, one, and two are utilized for training, and three and four for evaluation.

## Computational setup

Despite utilizing multiple participants, we are constrained by a limited amount of training data and consequently encounter the challenge posed by the Transformer’s weak inductive bias. Therefore, pre-training, data augmentation, and model regularization have been incorporated into the training process to successfully train the model and prevent overfitting. This approach has been applied successfully in the context of ViT by Steiner et al.^50^, where the authors demonstrated that it can lead to improvements akin to scaling up the dataset size by an order of magnitude.

**Fig. 2:**
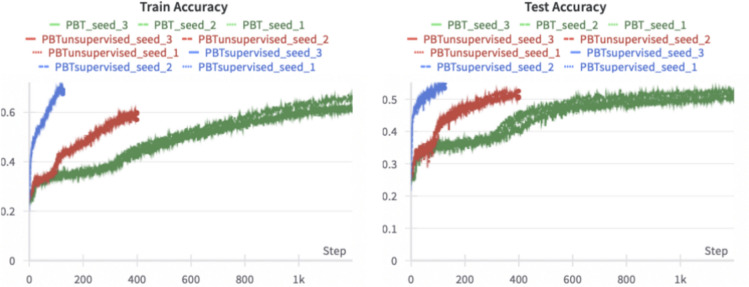
Comparison of training behaviors for PBT when trained from scratch, with self-supervised pre-training, and with supervised pre-training on the BCI Competition IV 2a data. The illustration shows that the model with self-supervised pre-training achieves the same test accuracy with only one-third of the fine-tuning compute budget required for training from scratch. However, the model with supervised pre-training outperforms both in terms of accuracy and compute efficiency.

The following experiments are performed on a single Tesla V100 GPU using PyTorch^51^. The computation times are approximately 11 h for supervised pre-training and 9 h for self-supervised pre-training. During fine-tuning for BCI Competition IV 2a, training times vary depending on the number of iterations. Training from scratch takes approximately 80 min while starting from self-supervised pre-training reduces the time to 25 min. With supervised pre-training, it takes only 10 min to complete the fine-tuning process (see Fig. 2).

### Pre-processing

Due to the non-invasive nature of EEG recordings, sensors are attached to the scalp, resulting in signals with a substantial amount of noise. To increase the signal-to-noise ratio, we apply the Infinite Impulse Response bandpass filter from the MOABB package. This filter narrows down raw EEG signals effectively and focuses on the frequencies of interest.

The best results are achieved within the frequency range of 8 Hz to 45 Hz, aligning well with established theory^52^. Notably, within the considered spectrum, prominent alpha waves (8-12 Hz) are observed, indicating a relaxed state, while beta waves (12-35 Hz) suggest a busy, active mind state of the participants. Additionally, gamma waves ($\ge$ 35 Hz) are noted, indicating a high concentration level in the subjects.

We find it important to preserve the amplitude ratio within a session and on a channel level. Therefore, the data are normalized to zero mean and unit variance, applying channel-wise Z-score, over all trials from the same recording session, $\frac{x_c - \mu _c}{\sigma _c}$, where $x_c \in \mathbb {R}^T$ denotes a single trial of channel $c \in \{1, \dots , C\}$. Here, $\mu _c$ represents the mean and $\sigma _c$ the standard deviation calculated over all trials from the same recording session and channel *c*. Note that the normalization is applied separately to the training and evaluation data.

### Data augmentation

We use data augmentation to expand the number of training samples. The effects of each individual method on PBT are analyzed in Section “Results”. For further discussion on EEG signal data augmentation, we refer to Mohsenvand et al.^53^. The listed techniques below are implemented on a per-trial level.

**Gaussian Noise.** We increase the noise contained in the EEG signals due to the recording by adding Gaussian noise with a mean of $\mu =0$ and a standard deviation of $\sigma = 0.1$.

**Direct Current Shifts** are applied by adding a constant vector of a normally distributed random number with $\mu =0$ and $\sigma = 0.1$ to each input.

**Amplitude Scaling.** The signal intensity is weakened or strengthened by multiplying a normally distributed number with $\mu =1$ and $\sigma = 0.1$ to the inputs.

**Time Shifts** are implemented by cropping the input time window to a smaller size and shifting this window to a random starting point in each training epoch. Given that the temporal order of the tokens remains unchanged, the positional embedding is unaltered.

### Model configuration

For our model, we select a patch dimension of $D=64$, resulting in $d_{model}=128$. The hidden size of the four Transformer blocks is configured to be 512, with four self-attention heads each, resulting in a total of approximately 884,000 parameters. We input two-second windows into the Transformer. Consequently, the signal is divided into eight tokens per channel. We use AdamW as an optimizer^54^. Weight decay is decoupled and we use a value of 0.01 for all weights, excluding those associated with bias, weights belonging to LN, the positional embedding, and the classification head. No weight decay is applied to the mentioned exceptions. Additionally, we set the values of $\beta _1$ and $\beta _2$ for AdamW to 0.9 and 0.95, respectively, and clip the gradient at a global norm of 1. During the warm-up training phase, we linearly increase the learning rate until it reaches a maximum value of $lr_{max}=3 \cdot 10^{-4}$, followed by a cosine decay schedule to the minimum of $lr_{min}=3 \cdot 10^{-5}$. Since the size of the training data is limited, a training epoch is one iteration of all training data. We implement dropouts^55^ with a rate of $P_{drop}=0.1$, following a similar approach as in the original Transformer model.

### Pre-training

The flexible architecture of PBT enables the model to contextualize a wide range of sensor arrangements, facilitating the processing of diverse datasets. Through the attention mechanism, PBT can effectively handle an arbitrary number of patches and therefore EEG channels. Information such as channel position and the chronological order of patches is incorporated through trainable positional embeddings. These embeddings consist of a limited number of parameters, which can be adapted or trained from scratch for the new sensor arrangement if necessary. Meanwhile, the vast number of weights is independent of the patch position and remains unchanged. To increase robustness, we leverage the flexibility of PBT and integrate five different motor imaginary datasets (see Section “Data desciption”) for self-supervised and supervised pre-training. The batches are randomly selected independently of the dataset, resulting in an unequal number of channels. For each trial, we randomly sample the included EEG channels, in addition to the time shifts, as data augmentation during pre-training.

#### Self-supervised pre-training

For the self-supervised pre-training, we follow the approach of BERT^25^, where randomly manipulated tokens are reconstructed. However, we need to ensure that the reconstruction task is not trivial. EEG signals are recorded as the voltage difference between a sensor and a reference sensor, as described in Mohsenvand et al.^53^. As a result, the signal from a sensor positioned spatially between two other sensors can be approximated as the mean of the two adjacent sensors. Due to this technical property of EEG-signals, we randomly manipulate patches along the time dimension with a probability $P_{\text {MASK}}= 0.3.$ The patches selected for manipulation are replaced by a mask in $80\%$ of instances, by a randomly selected patch from the current batch in $10\%$ of cases, and remain unchanged in another $10\%$. The percentages used here are consistent with those in the original BERT paper.

Furthermore, we observed that PBT operates on a latent frequency space, as discussed in Section “Inspection of the Patched Brain Transformer”. To address this, we incorporate an extra linear projection as the final layer to transform the Transformer outputs into the reconstructed EEG signal. The reconstruction loss, defined as $\mathcal {L} = 1 - \frac{x_p \cdot \hat{x}_p}{||x_p|| \cdot ||\hat{x}_p||}$, is calculated between Transformer outputs from manipulated patches $\hat{x}_p$ and the corresponding original patches $x_p$. The supervised model achieves higher accuracy than models trained from scratch or with self-supervised training. Therefore, we recommend using the self-supervised model only in specific cases, such as when the computational or time budget for fine-tuning is limited.

#### Supervised pre-training

In order to pre-train PBT in a supervised manner, we use the model and the labels of the pre-training datasets. To encourage the model to learn invariant representations with respect to subjects and recordings, we amalgamate appropriate classes across the datasets.

The chosen hyperparameters are the ones already described in Section “Model configuration”, with the following exceptions. We increase the weight decay factor for the classification head to one. This adjustment is crucial, because we reinitialize this layer before the fine-tuning step, as the classes between pre-training and fine-tuning do not align. The model is trained for 600 epochs with a warm-up phase of 100 epochs.

#### Semi-supervised pre-training

In our semi-supervised pre-training approach, we combined self-supervised and supervised pre-training. However, since we used the same data for the self-supervised part as for the supervised part, the results fell short compared to using solely supervised pre-training. Therefore, we do not further pursue this approach in this work, leaving it open for future research.

#### Fine-tuning

For the fine-tuning of PBT, we transfer the weights of the linear projection, positional embedding, and Transformer encoder from either self-supervised or supervised pre-training. In both cases, we reinitialize the classification head according to the number of classes contained in the fine-tuned dataset. The number of epochs for the warm-up phase and training depends on the dataset and the pre-training methods applied.

## Results

The results contain an examination of data augmentation, the evaluation of the impact of pre-training, and a comparison with other models in multi-participant classification tasks. In this section, we examine data augmentation, evaluate the impact of pre-training, and compare our model with others in multi-participant classification tasks.

### Comparison to other models

To ensure a fair comparison of results and maintain consistency, all models are trained and evaluated using a two-second-long time segment. For PBT, the training includes time shifts as data augmentation. In the evaluation phase, the segments are the same as for the competitive models, fixed at the middle of the experimental duration. As detailed in Section “Data description”, we assess the models on two datasets, one with two classes and the other with four classes, respectively. The data splits are derived from BCI Competition IV, and hyperparameter tuning is confined to the training data. Additionally, we employ identical hyperparameters for fine-tuning on both datasets, adjusting only the number of training epochs based on the dataset size. The same pre-training weights are utilized for both fine-tuning datasets. In Table 2, we provide evaluation results, representing the mean and standard deviation of three reproducible runs with random seeds $\{1, 2, 3\}$.

**Table 2:** Comparison of the accuracies and the number of parameters of the Patched Brain Transformer with leading reproduced models in the multi-participant setup.

Model	Number of parameters	BCI Comp. IV2a (4 Classes)	BCI Comp. IV2b (2 Classes)
PBT (no pre-training)	884K	51.43 ± 0.49%	76.58 ± 0.16%
**PBT (supervised pre-training)**	884K	**53.96 ± 0.70%**	**78.13 ± 0.51%**
PBT (self-supervised pre-training)	884K	51.21 ± 0.93%	76.86 ± 0.22%
EEGNet^11^	2.5K	52.44 ± 0.48%	76.35 ± 0.16%
DGCNN^56^	72.5K	38.11 ± 0.376%	73.28 ±0.11%
ATCNet^13^	115K	47.37 ± 0.01%	74.41 ± 0.01%
BIOT (no pre-training)^33^	3.3M	31.93 ± 0.71%	71.28 ± 0.49%
BIOT (self-supervised pre-training)^33^	3.3M	36.83 ± 1.08	75.80 ± 1.52%
LaBraM Base (no pre-training)^14^	5.8M	26.45 ± 0.08%	57.93 ± 3.18%
LaBraM Base (self-supervised pre-training)^14^	5.8M	27.60 ± 0.66%	67.75 ± 1.37%

Best accuracy values are in bold.

We compare our model against representatives of different architecture concepts. Among the CNN models, we compared it to EEGNet, a well-known model for EEG classification. The reported results were obtained using the implementation from the Braindecode package^57^. Additionally, we selected the ATCNet and DGCNN implementation from TorchEEG^58^ as it demonstrates state-of-the-art accuracy in single-participant motor imagery classification^59^. It is essential to note that the reported accuracy results, such as those available on papers with code, are based on distinct evaluation methodologies, making direct comparisons difficult. BIOT represents models with a CNN tokenizer in conjunction with a Transformer, while LaBraM applies a ViT to FFT-transformed EEG samples. The code and pre-trained models from BIOT^60^ and LaBraM^61^ are loaded from their respective GitHub repositories as referenced. Since only the weights for LaBraM Base are released and the pre-training is not reproducible, we employ the smallest model (LaBraM Base) from the LaBraM line of models.

The results from PBT outperform those from ATCNet and DGCNN on both datasets. While EEGNet achieves slightly superior results on the BCI Competition IV 2a dataset compared to PBT with no pre-training, the performance on BCI Competition IV 2b is similar for both models. We attribute this to the smaller size of EEGNet compared to PBT, allowing it to benefit from the limited trials per class in BCI Competition IV 2a. In our experiment, we did not observe an accuracy advantage from self-supervised pre-training. However, we did notice accelerated training on BCI Competition IV 2a, see Fig. 2. This suggests that self-supervised pre-training learns relationships between channels, which is particularly beneficial for datasets with multiple channels. The overall best results are achieved by PBT in combination with supervised pre-training.

### Impact of data augmentation

We evaluate the impact of the data augmentation techniques described in Section “Data augmentation” by comparing the results of PBT models trained from scratch on both fine-tune datasets. On the dataset from BCI Competition IV 2a, which has fewer trials per class, we achieve prediction results beyond the chance level only when employing time shifts as data augmentation. For all other approaches, PBT immediately overfits on the training data, resulting in no general prediction capabilities. Table 3 presents the accuracy results on the data from BCI Competition IV 2b. We randomly chose $30\%$ of the BCI Competition IV 2b data for evaluation. The aforementioned subset was kept fixed for all runs. The accuracy difference compared to the results presented in Table 2 comes from different data splits. Subsequently, we trained PBT for all methods on the same fractions of the remaining data. These results confirm the findings on the other dataset: employing time shifts for data augmentation achieves the best accuracy results on the evaluation data. Furthermore, including Gaussian noise as data augmentation slightly improves the results compared to no augmentation. However, the joint application of time shifts and Gaussian noise does not improve the sole application of time shifts on both datasets.

**Table 3:** Comparison of the accuracy results for PBT with different data augmentation techniques across the various sized fractions of the BCI Competition IV 2b dataset. For brevity, we present the results rounded to the nearest whole percentage.

Augmentation	Training data fraction			
Method	10%	17.5%	35%	70%
No augmentation	54%	55%	60%	63%
Gaussian noise	55%	57%	62%	65%
DC shifts	54%	55%	60%	63%
Amplitude scaling	54%	55%	60%	63%
Time shifts	**70%**	**70%**	**71%**	**73%**

Significant values are in bold.

Note that LN mitigates the impact of amplitude scaling and DC shifts applied beforehand. However, it is important to recognize that every LN operation implicitly applies amplitude scaling and DC shifts to achieve mean zero and unit variance.

### Inspection of the Patched Brain Transformer

#### Linear projection

**Fig. 3:**
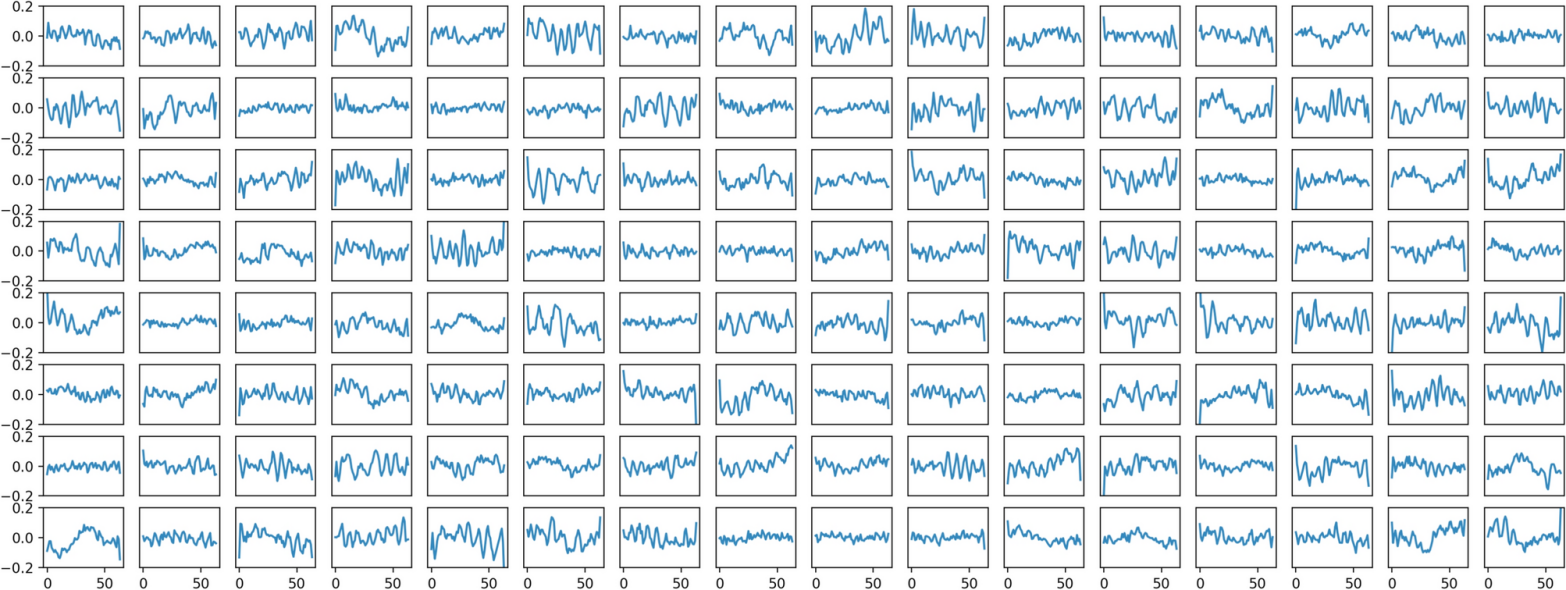
Temporal Filters. Each of the subplots visualizes the learned weights of the linear projection layer applied to each token. The temporal dimension of the token is shown on the x-axis and reveals periodical structures. This indicates that the Transformer operates on a frequency space.

 By analyzing the weights of the linear embedding, i.e. the first layer of PBT, see Fig. 3, we observe periodic structures that we interpret as frequency filters. Based on time windows with a size of 64 samples per patch and a sampling frequency of 250 Hz, each patch corresponds to 0.256 s. Consequently, we anticipate the frequencies as four times the number of cycles per patch. Most of the learned filters are in the high-frequency range between 20 and 40 Hz, which corresponds to the beta and gamma waves and, to higher concentration levels for the participants^52^. Comparable filters are learned in the first convolutional layers of EEGNet^11^. The filters from the linear projection show no evidence for our assumption that each attention-head from the MHSA is processing a particular frequency range.

#### Positional embedding

**Fig. 4:**
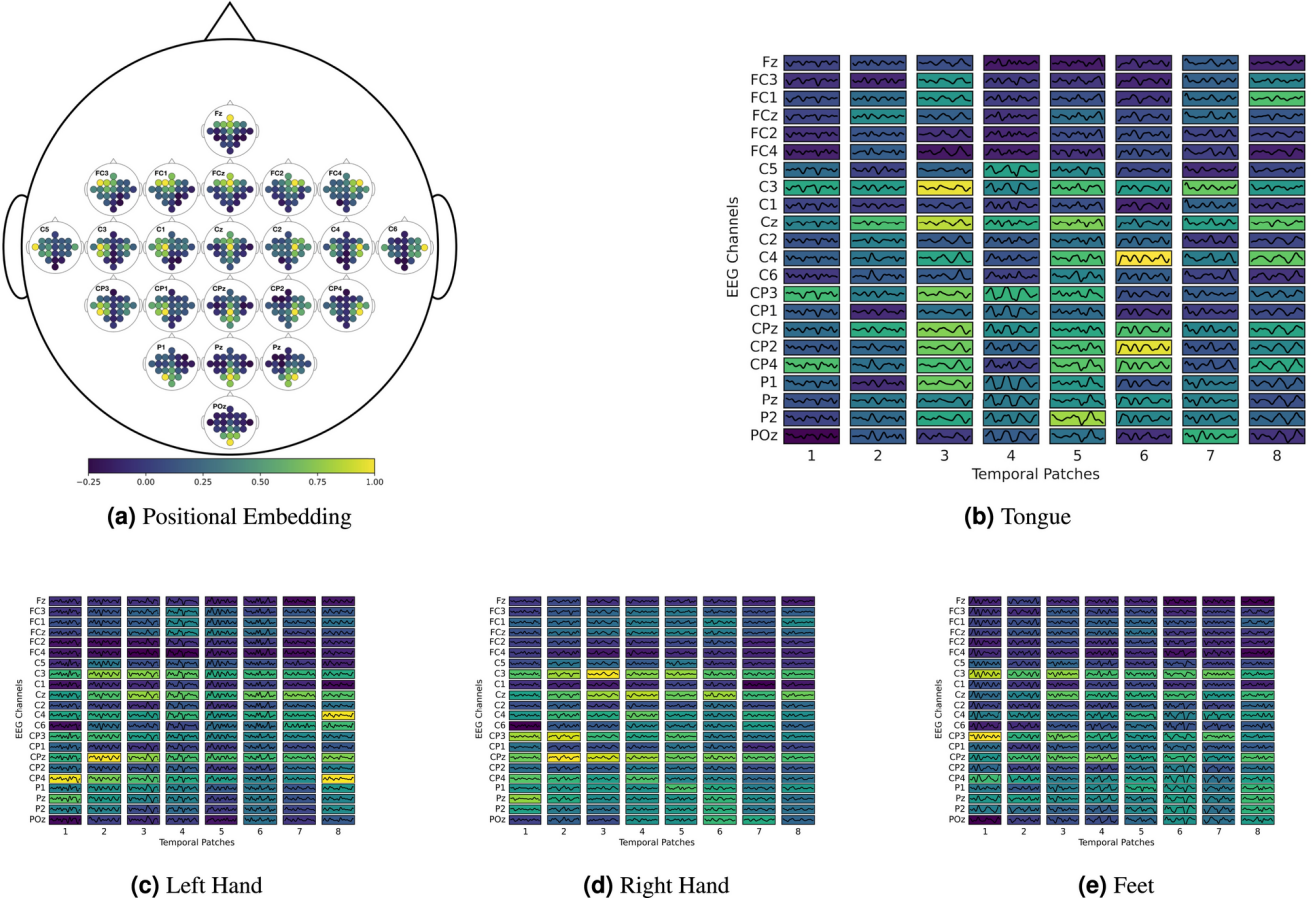
(**a**) Visualization of the learned spatial sensor positions on the BCI Competition IV 2a dataset. Each sensor is depicted as a subplot, illustrating the similarity of the learned positional embedding to all others. (**b**)–(**e**) Attention Rollout for PBT on BCI Competition IV 2a dataset. Tokenized example trials of the first subject, featuring class in the caption. We display EEG signals on a background where brighter colors represent the higher importance of each patch for classification.

We deploy a learned positional embedding rather than an explicitly given one since the spatial-temporal position of the tokens is not clear. Figure 4a illustrates the spatial relation of the learned positional embedding for the 22 channel montage. Each subplot represents the spatial position of this sensors on the scalp. The dots on the color scale in each subplot indicate the cosine similarity, $\frac{x \cdot \hat{x}}{\Vert x\Vert \Vert \hat{x}\Vert }$, between the positional embedding of that sensor and the embeddings of all sensors. Consequentially, each subplot contains a dot that represents the similarity to itself, which is 1, illustrated as bright yellow. In general, the positional embedding demonstrates greater similarity to sensor positions that are spatially closer. However, we also notice a symmetric pattern along the left and right hemispheres of the brain in the central region (C1–C6). This region is associated with the motor cortex, particularly *C*3 being linked to the right hand and *C*4 to the left hand^62^. Further discussion on how this relates to brain activity can be found in the “Discussion” Section.

#### Attention rollout

In Fig. 4b–e, we investigate the attention mechanism of PBT with attention rollout^63^. This method, based on the recursive multiplication of the attention weight matrices, comprehends the attention between tokens. We note that trained Transformer attends both spatial and temporal dimensions integrating information across the entire recording. This technique could serve as a tool for further research in the field of neuroscience as it highlights the tokens that are important for classification. However, this is beyond the scope of this work.

## Discussion

We proposed the *Patched Brain Transformer*, a novel attention-based model for EEG decoding. The model architecture is specifically designed to address key challenges in EEG decoding, namely noise, data scarcity, and temporal-spatial dependencies.

We mitigate noise in the data by applying a bandpass filter during pre-processing, which limits the EEG signal to relevant frequencies. Additionally, as demonstrated in Section “Impact of data augmentation”, layer normalization further reduces the influence of noise on the predictions. While the effect of normalization on noise in EEG data has not been extensively studied in prior research, normalization is widely used in machine learning to stabilize training.

The PBT model employs a self-attention mechanism on linearly embedded patches of raw EEG data, enhanced by positional embeddings. This design allows the model to handle varying numbers of input tokens, offering crucial flexibility with respect to channel count and input duration. Such flexibility is essential for effective pre-training across diverse datasets, as EEG recording paradigms often vary between experiments. Unlike CNN-based models like EEGNet or Transformer models with CNN tokenizers, such as ATCNet, our model can seamlessly adapt to varying data layouts. This adaptability enables effective pre-training on diverse datasets, which helps address data scarcity during fine-tuning. Additionally, our experiments demonstrate that using time shifts in PBT as a data augmentation technique effectively enhances the training process, further compensating for limited training data.

The PBT model is also designed to learn temporal-spatial dependencies in EEG data without explicit guidance. Given that Transformers are inherently position-invariant, each token is updated with a learned positional embedding to convey temporal and spatial information within the EEG sequence. The self-attention mechanism enables every token to attend to every other token, capturing global temporal and spatial dependencies across the entire input. This capability is particularly valuable for EEG data, where relevant information may be distributed across different temporal points or spatial electrodes, allowing the model to recognize both short-term and long-term patterns in EEG signals without being constrained by the distance between tokens. In contrast to CNNs, where spatial and temporal dependencies are built up gradually through layers, the Transformer’s self-attention mechanism allows PBT to capture these dependencies across the entire EEG sequence immediately and adaptively.

We evaluated the performance of the PBT on two well-known datasets from the BCI Competition against recent models in brain decoding. The comparison demonstrate that the model not only matches but surpasses state-of-the-art models on motor imagery tasks while utilizing fewer parameters. This result suggests that our model achieve superior performance on similar decoding tasks like emotion recognition, classification of focus states and control for robotics or games. The best results were achieved with a supervised pre-trained version of PBT, combined with data augmentation and model regularization. Supervised pre-training is performed on five motor imagery datasets with different recording paradigms, enabled by a flexible model design in terms of the number of EEG channels and recording duration. The incorporation of data augmentation techniques, such as time shifts, significantly enhanced the model’s robustness and generalizability across participants. The positive impact of augmentation highlights its importance in training deep learning models on limited EEG data, suggesting that such techniques should be a standard consideration in future EEG machine learning studies. Furthermore, the training process includes model regularization, namely weight decay, excluding the final linear layer.

In contrast to prior approaches that employ CNN as a tokenizer, our method facilitates only one linear projection layer. Figure 3 reveals that this layer learns similar frequency filters to convolutional networks, e.g., EEGNet, and justifies the omission of the CNN as a tokenizer. The learned filters extract brain frequencies associated with higher levels of participant concentration.

In Fig. 4a, we illustrate the learned positional embeddings in the spatial domain. As expected, the visualization of the positional embedding shows similarities between spatially related sensors. Interestingly, EEG sensors located in the central area $(C1-C6)$ exhibit relationships between corresponding sensor positions on the left and right hemispheres of the brain, despite the lack of spatial proximity. The central region is associated with the motor cortex, particularly *C*3, which is linked to the right hand, and *C*4, to the left hand. Since the model is trained to discriminate movements between the hands, and the signals for moving the right and left hands are similar, except for the position where the signals are recorded, this relationship is observed.

Figure 4b–e shows that most of the intensities are concentrated in the *C*3 (located over the left motor cortex, corresponding to the right side of the body.), *C*4 (located over the right motor cortex, corresponding to the left side of the body), and *Cz* sensors (positioned along the mid-line of the motor cortex, often associated with bilateral motor control). These electrodes that correspond to motor function are primarily located over the motor cortex, which is involved in controlling voluntary movements.

The potential applications of our model extend beyond MI classification. Enhanced EEG decoding could facilitate early detection of neurological disorders and mental health conditions. The model’s ability to decode EEG signals accurately with small data and computational resources positions it as a valuable tool for real-world applications.

While the capabilities of *Patched Brain Transformer* are promising, numerous challenges persist. One such challenge involves further exploration of the transferability and scalability of pre-training across different classification tasks. Additional focus could be laid on investigating the internal representations in PBT and their connection to underlying neural processes. Achieving these future directions would enable the development of more generalized and adaptable EEG-based models for motor imagery classification.

Overall, the Patched Brain Transformer represents a step forward in EEG decoding, offering an adjustable, efficient, and interpretable machine learning approach. The model demonstrate strong performance and alignment with neuroscientific knowledge, particularly in motor imagery tasks. By leveraging advanced attention based techniques and focusing on practical applications, this study contributes to computational neuroscience, highlighting its potential for broader applications in medical diagnostics.

## Data Availability

All datasets used in pre-training and fine-tuning are publicly accessible. All datasets used in pre-training and fine-tuning are publicly accessible. Table 1 provides a concise summary of the datasets with citations to the publications for each dataset, which offer more detailed descriptions of the datasets and access information. For example, the fine-tuning data is provided by Graz University of Technology for BCI Competition IV^7^, available at^64^. Furthermore, we utilize the MOABB (Mother of All BCI Benchmarks) library^65^ to access the data conveniently.

## Code availability

The code for the models, the training pipeline for pre-training and fine-tuning, as well as the trained models, is publicly available on GitHub https://github.com/timonkl/PatchedBrainTransformer.
